# Oral and maxillofacial considerations in Gardner’s syndrome: a report of two cases

**DOI:** 10.3332/ecancer.2016.623

**Published:** 2016-02-24

**Authors:** Debora Lima Pereira, Paulo Andre Carvalho, Maria Isabel Waddington Achatz, AndreCaroli Rocha, Giovana TardinTorrezan, Fabio Abreu Alves

**Affiliations:** 1 Department of Stomatology, A. C. Camargo Cancer Centre, São Paulo, SP 01509-900, Brazil; 2 Department of Oncogenetics, A. C. Camargo Cancer Centre, São Paulo, SP 01509-900, Brazil; 3 Genomicsand Molecular Biology Laboratory, A. C. Camargo Cancer Centre, São Paulo, SP 01509-900, Brazil; 4 Department of Stomatology, São Paulo University, São Paulo, SP 01509-900, Brazil

**Keywords:** familial adenomatous polyposis, Gardner’s syndrome, maxillofacial, osteomas

## Abstract

Gardner’s syndrome (GS) is a genetic disorder characterised by intestinal polyps, multiple osteomas, and soft-tissue tumours. Dentists play an important role in the syndrome diagnosis considering that craniomaxillofacial osteomas are a major criteria for Gardner’s syndrome diagnosis. This study aimed to describe the main stomatological manifestation of GS and the importance of dentists in its diagnosis. Two patients presenting GS were evaluated. The first one had two osteomas in the mandible and GS was suspected. The colonoscopy confirmed the presence of polyposis and a prophylactic proctocolectomy was performed. The other patient had a late-stage diagnosis of GS and developed a rectum adenocarcinoma. The presence of craniomaxillofacial osteomas are a hallmark of the disease. Early-stage GS diagnosis may enable early diagnosis and preventive strategies in carriers. Other dental abnormalities, such as supernumerary teeth, hypercementosis and odontomas, can also be observed.

## Introduction

Gardner’s syndrome (GS) is an autosomal dominant inherited disorder described by Gardner in 1953 that predisposes individuals to a high risk of developing colonic polyposis, colorectal cancers, multiple maxillofacial osteomas and mesenchymal tumours [1]. It is a variant form of familial adenomatous polyposis (FAP), a genetic disorder marked by a mutation in the band of chromosome 5q21-q22, the adenomatous polyposis coli locus (APC gene) [1, 2, 3]. The prevalence is 1 per 8300 to 1 per 14,000 newborns with a slight predilection for women [2].

Although osteomas arise during puberty, dental alterations (congenitally missing teeth, hypercementosis, odontomas, dentigerous cysts, impacted teeth, supernumerary teeth, fused or unusually long roots) may be evident even in childhood. These alterations precede the polyposis in around 10 years [1, 4]. The polyps begin to develop at age 20 and almost 100% will suffer malignant transformations between 30 and 50 years old. Consequently, the early diagnosis of maxillofacial alterations of GS established by dentist may enable an early diagnosis of the syndrome [1, 5, 6]. Other manifestations of GS include hypertrophy of retinal pigmented layer (90% of cases), fibromas, leiomyomas, lipomas, meningiomas, epidermoid cysts, papillary thyroid cancer, osteosarcoma, and chondrosarcoma [1].

In this study, the oral manifestations of the GS were documented in two cases. In addition, the importance of early detection of the syndrome by dentists was also emphasised.

## Case Report

### Patient 1

An 18-year-old girl was referred to our department for abnormal mouth opening for 8 months. Her medical history revealed a surgery to remove an epidermoid cyst in the knee 4 years before the first evaluation. In addition, the patient was also complaining of abdominal pain and frequent diarrhoea for 18 months.

**Figure 1.**
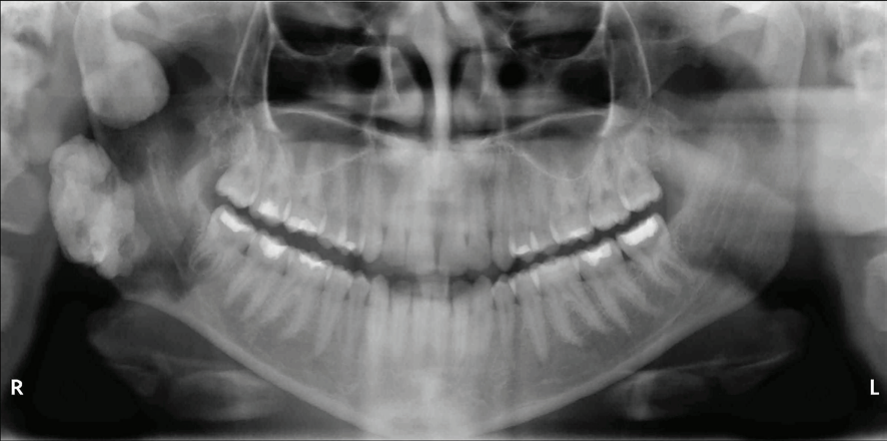
Panoramic examination of patient 1. The examination shows radiopaque lesions just inferior to the right mandibular condyle and the mandible angle.

**Figure 2.**
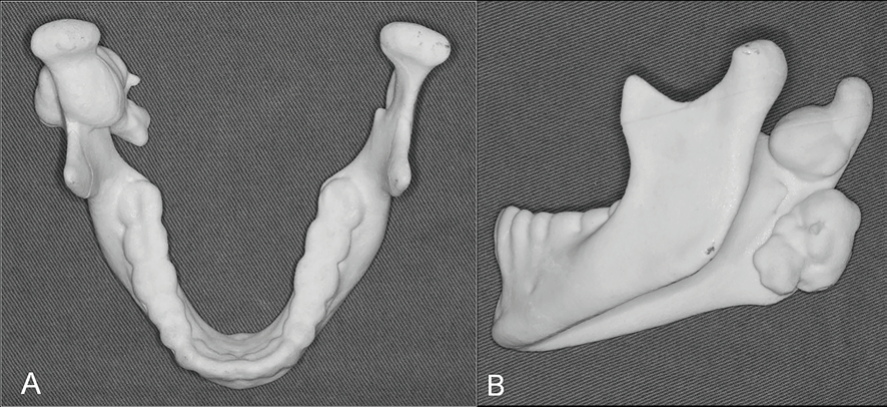
Computed tomography with solid prototype. Solid prototype in up (a) and lateral (b) view showing osteomas in the condylar region and mandible angle that were surgical removed.

The extraoral examination showed a fixed nodular swelling in right side of the mandible (angle region), and there was no alteration in the intraoral examination. Both panoramic radiograph (Figure 1) and computed tomography (CT) with solid prototype (Figure 2a, b) revealed two well-defined markedly radiopaque/radiodense lesions arising on the ramus (27 × 19 mm) and the mandible angle (23 × 15 mm). These lesions were very suggestive of osteomas and GS was hypothesised according to anamnesis and mandible lesions. A colonoscopy showed colonic and rectal polyposis. The biopsy showed multiple tubular adenomas with low-grade dysplasia. GS diagnosis was confirmed and the patient underwent a total proctocolectomy with ileal pouch anal anastomosis. Germline mutation in APC gene was found in the patient, who carried a deletion of five nucleotides, c.3927_3931delAAAGA (p.Glu1309Aspfs*4). Her family members were evaluated and none of them presented with GS.

Due to oral symptoms (temporomandibular junction pain and deviation of mouth opening), both osteomas were excised. After 23 months, the patient is asymptomatic.

### Patient 2

A 49-year-old woman was referred to our department to evaluate the occurrence of jaw abnormalities. Her medical history showed FAP diagnosis when she was 30 years old. The patient had developed adenocarcinoma in the rectum and an epidermoid cyst in the left subgluteus region at age 47. Familial history showed her father, one brother, three sisters, and three cousins with symptoms that were typical of FAP diagnosis. A germline mutation in APC gene (c.3880_3881delCA, p.Gln1294Glyfs*6) was found in this patient.

**Figure 3.**
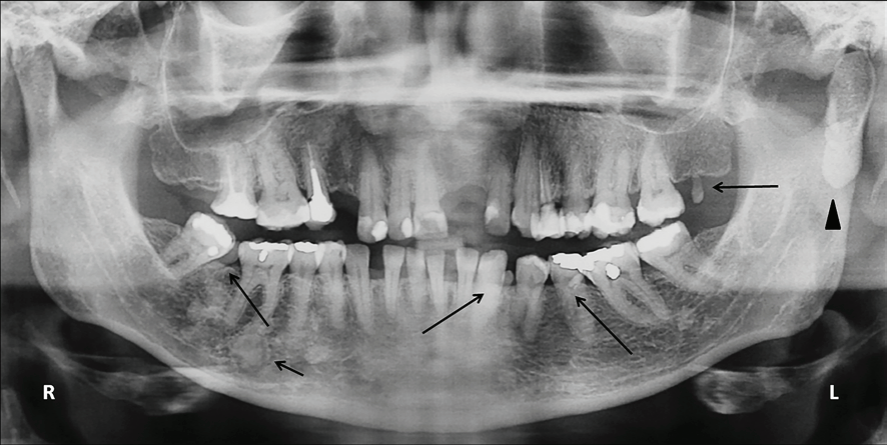
Panoramic radiographic patient 2. The presence of osteoma in the left condylar region (arrow head). Note supernumerary teeth in the mandible and maxilla (long arrow). Osseous dysplasia can also be observed throughout the mandibular body (short arrow).

Her dental history revealed the excision of the left superior incisor impacted in an odontoma. On intraoral examination was observed three supernumerary teeth in mandible and one in maxilla. The panoramic radiography revealed an osteoma in the left condylar region and multiple diffuses radiopaques lesions on the lower teeth periapices, which were diagnosed as osseous dysplasia (Figure 3). The patient and her relatives are regularly followed.

## Discussion

FAP is characterised by germline mutations in APC gene [2]. Gardner’s syndrome is part of a group of disorders that include familial polyposis coli (simple form) and Turcot’s syndrome (SNC compromising) related to adenomatous polyposis [4]. In general, polyps may affect the entire gastrointestinal tract and most start to develop during puberty. The occurrence of malignant transformation in GS occurs in almost 100% of the cases around fourth and fifth decades of life [1, 6]. Due to high potential of malignant transformation, the preventive colostomy is recommended to patients with countless polyps as the only method to avoid the colon cancer [6]. In this study, we present two cases of GS, including a *de novo* mutation carrier and a case within extensive familial history of FAP.

Osteoma is an important non-odontogenic alteration related to GS and the mandible is more affected than the maxilla, but it can occur in any region of the skeleton. Its presence is fundamental to the GS diagnosis and 26–46% of the patients show three or more lesions [2, 6]. Young patients with osteoma should be evaluated promptly for other symptoms of the syndrome. In general, the osteomas are treated by surgery, which is indicated only to symptomatic cases or aesthetic purposes and its recurrence is rare. Beyond osteoma, some patients can present diffuse sclerosis throughout the mandibular body [6, 7]. In the present study, surgery was performed only in the first patient, who had two large osteomas causing deviation of mouth opening and pain.

Radiologically, osteomas present as a well-defined radiopaque mass with density similar to normal bone and, sometimes, it can be pedunculated. The differential diagnosis includes exostoses, osteochondroma, osteoblastoma, osteosarcoma and complex odontoma. In general, exostoses have a limited growth and are located in marginal gingival and hard palate. Osteochondromas are composed of heterogeneous areas (radiopaque and radiolucent) and osteoblastoma and osteosarcoma present rapid growth [8]. In the present study, the patient 1 had 2 ovoid radiopaque lesions and osteomas were the main diagnosis hypothesis. This diagnosis was considered mainly due to the patient presenting with two synchronous lesions and the lesions above, except exostosis, are commonly single lesions.

The main odontogenic-related alterations/lesions in patients with GS are congenitally missing teeth, multiple ectopic teeth, retained deciduous teeth, hypercementosis, odontomas, dentigerous cysts, impacted teeth, supernumerary teeth, fused or unusually long roots [9]. The frequency of such abnormalities varies in literature from 17% to 75% [3, 6, 10]. The supernumerary teeth are generally located between normal teeth or impacted and show a small conical shape, as seen in our second patient who also presented odontoma and osseous dysplasia. There is no specific pattern in the number and type of odontogenic lesions. These alterations can be variable according to syndrome manifestation.

## Conclusion

In conclusion, dentists must play a decisive role in diagnosing patients with osteomas and referring to genetic counselling and surgical evaluation to determine the presence of FAP. This may enable early diagnosis of the syndrome and severe complications such as colon cancer.

## Conflicts of interest

The authors report no conflicts of interest.
